# Impact of rapid maxillary expansion on mouth-breathing children and adolescents: A systematic review

**DOI:** 10.4317/jced.58932

**Published:** 2021-12-01

**Authors:** Raquel-Harumi-Uejima-Satto Sakai, Maíra-Seabra de Assumpção, José-Dirceu Ribeiro, Eulalia Sakano

**Affiliations:** 1 Universidade Estadual de Campinas (Unicamp), Faculdade de Ciências Médicas, Departamento de Pediatria, Campinas, SP, Brazil; 2 Universidade Estadual de Campinas, Hospital das Clínicas, Departamento de Otorrinolaringologia, Campinas, SP, Brazil

## Abstract

**Background:**

Rapid maxillary expansion (RME) is an orthodontic procedure used to correct transverse maxillary deficiency. Due to the anatomical relationship between the palate and the nasal cavity, RME promotes an increase in nasal dimensions, which should hypothetically improve nasal respiratory function. Objective: This review aimed to systematically verify studies that assessed the effects of RME on nasal patency in mouth-breathing children and adolescents.

**Material and Methods:**

An electronic search was performed in the MEDLINE databases via OVID, Scopus and EMBASE. The terms were: “children and adolescents”, “rapid maxillary expansion” and “mouth breathing”. The search was conducted in October 2019, according to the criteria of the Preferred Reporting Items for Systematic Reviews and Meta-Analyses (PRISMA). The assessment of the quality of the studies was conducted by two evaluators, using the Fowkes & Fulton´s guidelines for critical appraisal of medical research.

**Results:**

475 titles were identified and 18 articles were selected. All of them showed high methodological quality, but without randomized clinical trials. The instruments evaluated were: teleradiography, frontal postero-anterior radiography, computed tomography, acoustic rhinometry and computed rhinomanometry.

**Conclusions:**

This review shows that RME promotes the enlargement of dental arches and of the nasal and maxillary structures, with improved mouth breathing in the short term. However, its long-term benefits could not be proved so far. More robust results of the effectiveness of RME in mouth breathing can be achieved with meta-analysis studies, with a consensual definition of the long-term follow-up period after RME.

** Key words:**Child, adolescent, maxillary expansion, palatal expansion, mouth breathing.

## Introduction

Mouth-breathing children often have a narrow upper arch with high palate. These reduced nasal and maxillary transverse dimensions may be related to the increase in nasal flow resistance, observed in these children (1).

Transverse maxillary deficiency (TMD), also called maxillary atresia, when corrected at an early stage, improves the child’s craniofacial and stomatognathic development, with beneficial effects on nasal breathing (2,3,4). The most frequently used orthodontic procedure for the correction of TMD is the rapid maxillary expansion (RME), which, through a fixed orthodontic expander positioned on the palate, causes the opening of the median palatal suture (5). Due to the anatomical relationship between the palate and the nasal cavity, RME promotes an increase in nasal dimensions, which should hypothetically improve nasal respiratory function (6-8).

Radiographic exams have proved the effects of RME on dental, nasal and maxillary structures (9). Three-dimensional CT imaging techniques have been increasingly used, with more accurate measurements, without overlapping structures of two-dimensional radiographs, but with a larger amount of radiation (0). International protocols are still being researched with the aim to optimize the amount of radiation used in volumetric cone-beam computed tomography (CBCT), especially in children and adolescents (11).

Several authors (12-14) report the immediate improvement in mouth breathing due to the increase in the nasal base with RME. However, some studies have not provided evidence of its stability in the long term (15,16). The evaluation of the respiratory function of the functional changes of children and adolescents mouth breathers (MB) allows these patients to be followed up, based on medical evaluations and complementary exams. The importance of evaluating these individuals is emphasized, with the possibility of respiratory function being reestablished with the early performance of ERM.

The present systematic review aimed to verify the effects of RME in MB (children and adolescents) on naso-maxillary structures and on nasal respiratory function, as well as to verify if RME remains unchanged in the long term.

## Material and Methods

-Search strategy

The search for scientific articles was conducted using the MEDLINE databases via Ovid, Scopus and EMBASE. The search included descriptors and their variant forms based on the Medical Subject Headings (MeSH), DeCs (Health Descriptors) and Emtree (Embase Subject Headings) that identified “rapid maxillary expansion”, “mouth breathing” and “children and adolescents”. Studies in English and French were selected, without date restrictions. The search was carried out from November 2018 to October 2019. The detailed search strategy with the terms used can be found in the APPENDIX.

-Study selection

**Figure 1:**
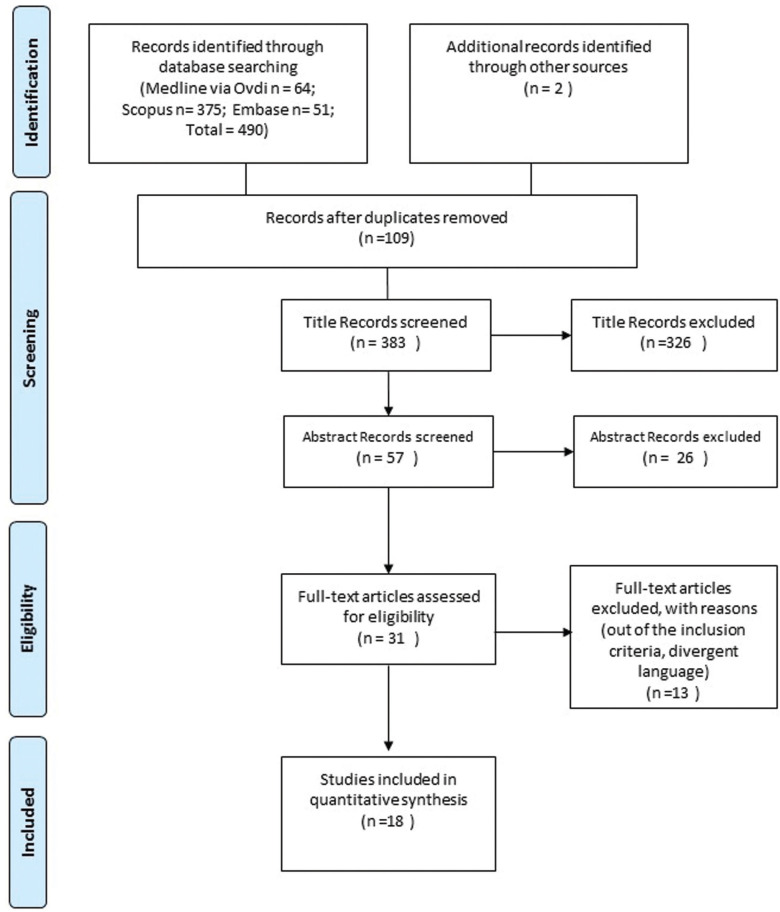
PRISMA.

The systematic review followed the Preferred Reporting Items for Systematic Reviews and Meta-Analyses (PRISMA) criteria (17). Two independent evaluators conducted the selection of the studies according to pre-established criteria. In case of disagreement, a third evaluator was consulted for a final decision (Fig. 1). At first, the duplicates were selected and removed, and then, the titles and abstracts were screened. Subsequently, the remaining articles were read in full, following the inclusion and exclusion criteria described below. Ultimately, a manual search of the references of the selected studies was performed.

-Inclusion and exclusion criteria 

The following inclusion criteria were used: (i) original studies; (ii) studies with mouth-breathing children and adolescents, which evaluated the effects of mouth breathing on nasal respiratory function using RME; (iii) cohort, controlled or randomized longitudinal studies; and (iv) studies published in full in English or French.

The following exclusion criteria were used: (i) book chapters, theses, dissertations, retrospective studies, editorials, letters, abstracts, comments, editorials, presentations in congress, symposia, seminars, round Table and debates, post scripts, patents, case reports and case series; (ii) studies that were not available in full on the internet even after contacting the authors; (iii) systematic reviews, literature reviews or meta-analyses; (iv) qualitative studies; (v) studies in which the RME procedure was performed only on nasal breathers and not on mouth breathers; (vi) studies without a diagnosis of nasal breathing pattern; (vii) studies without assessing nasal respiratory function before and after RME; (viii) studies that did not perform RME; and (ix) studies that included adult populations.

-Data extraction 

The following data were extracted from the selected studies: nationality, study design, sample size, age and sex of the study group and the control group and instruments evaluated (Table 1,  Table 1 cont). More detailed information such as objective of the study, main results and conclusions were summarized in Table 2,  Table 2 cont.,  Table 2 cont.-1,  Table 2 cont.-2.

**Table 1:**
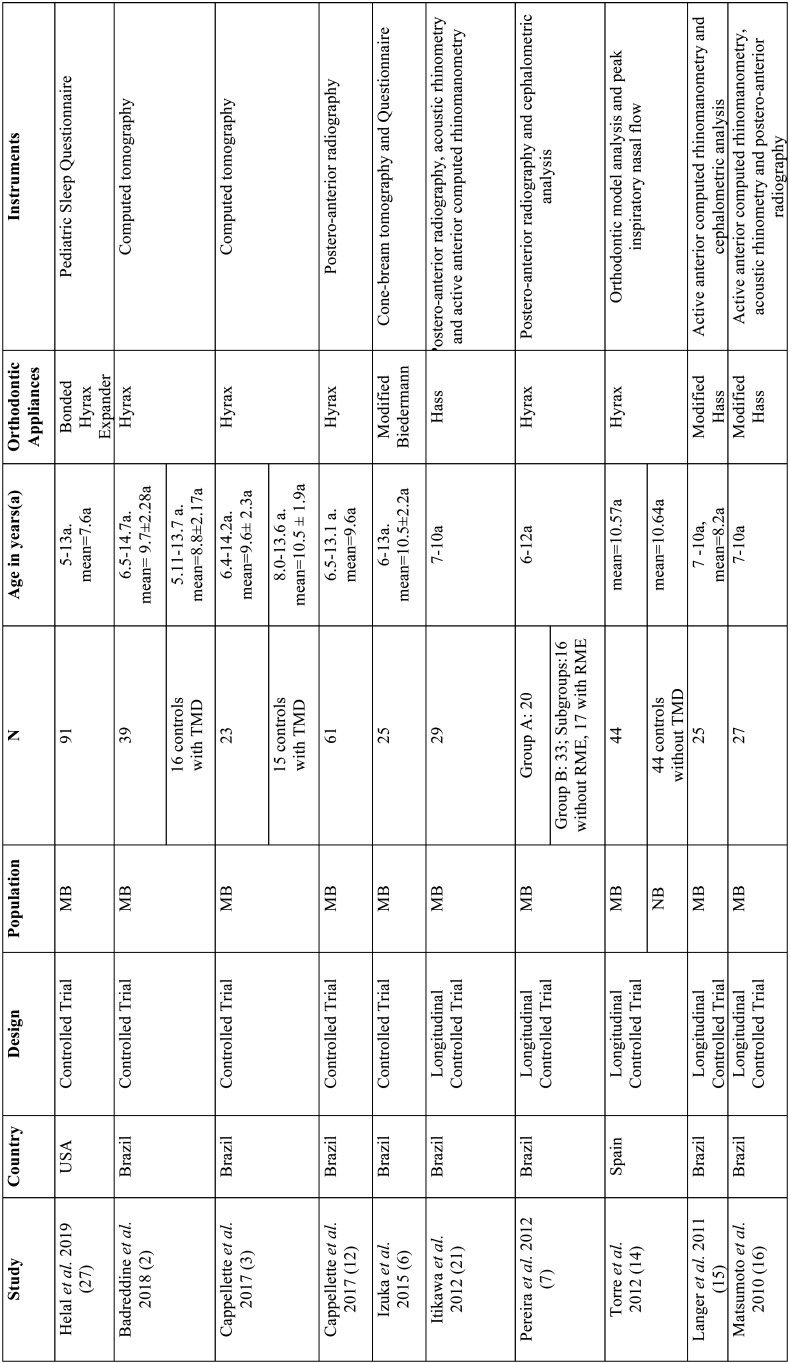
Analysis of samples, orthodontic appliances and instruments used in each study.

**Table 1 cont.**
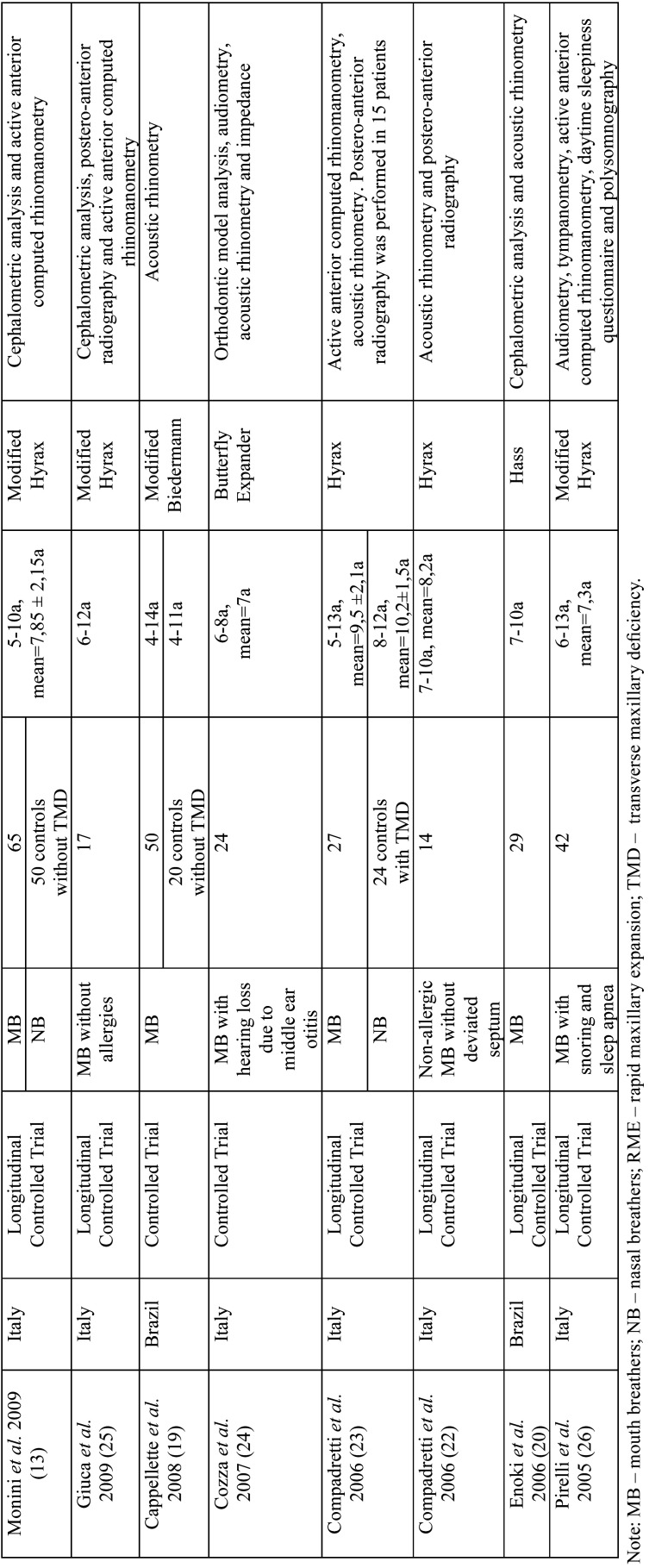
Analysis of samples, orthodontic appliances and instruments used in each study.

**Table 2:**
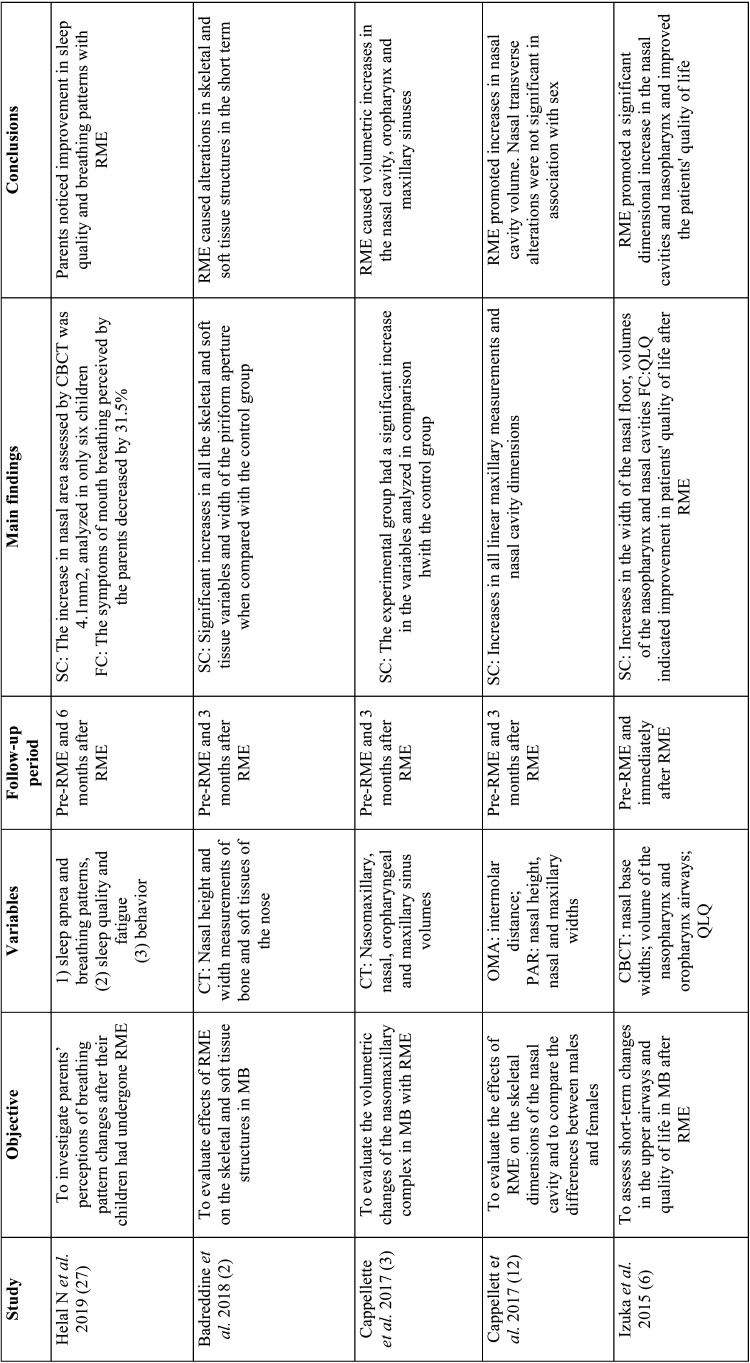
Analysis of objectives, variables, follow-up period, main results and conclusions.

**Table 2 cont.**
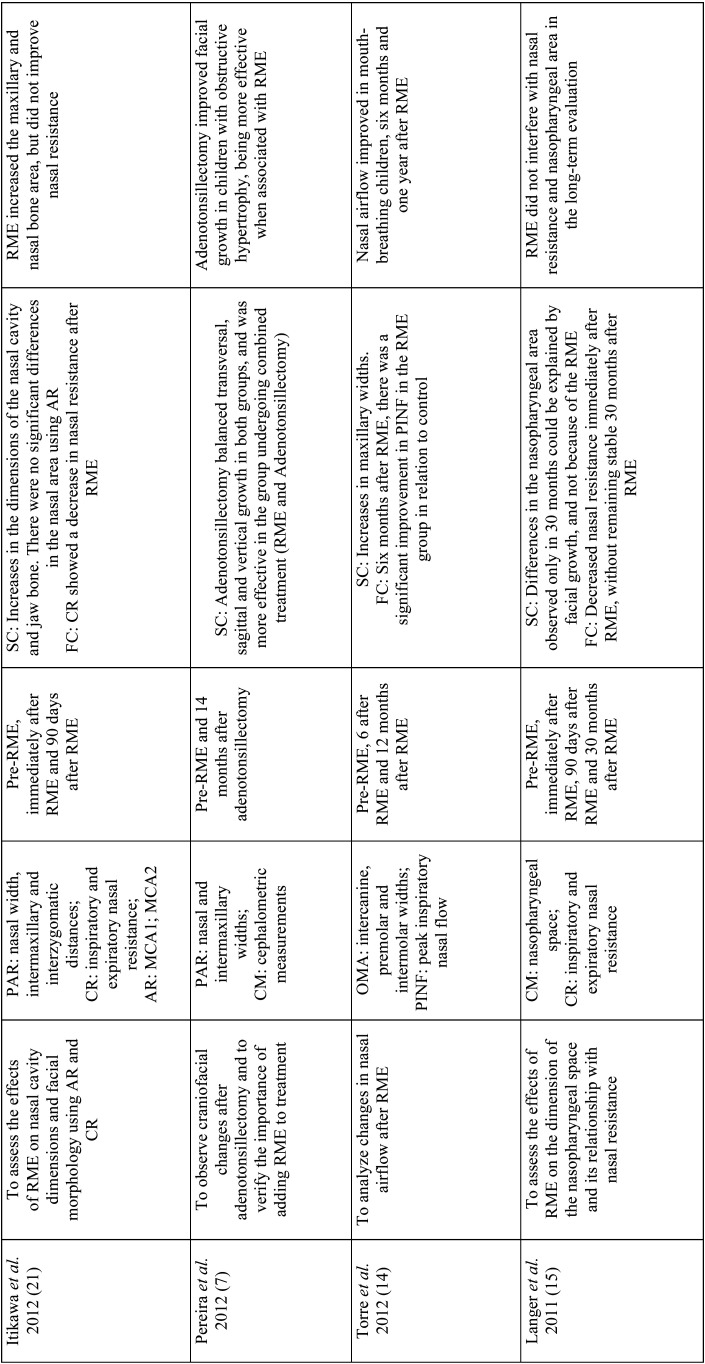
Analysis of objectives, variables, follow-up period, main results and conclusions.

**Table 2 cont.-1:**
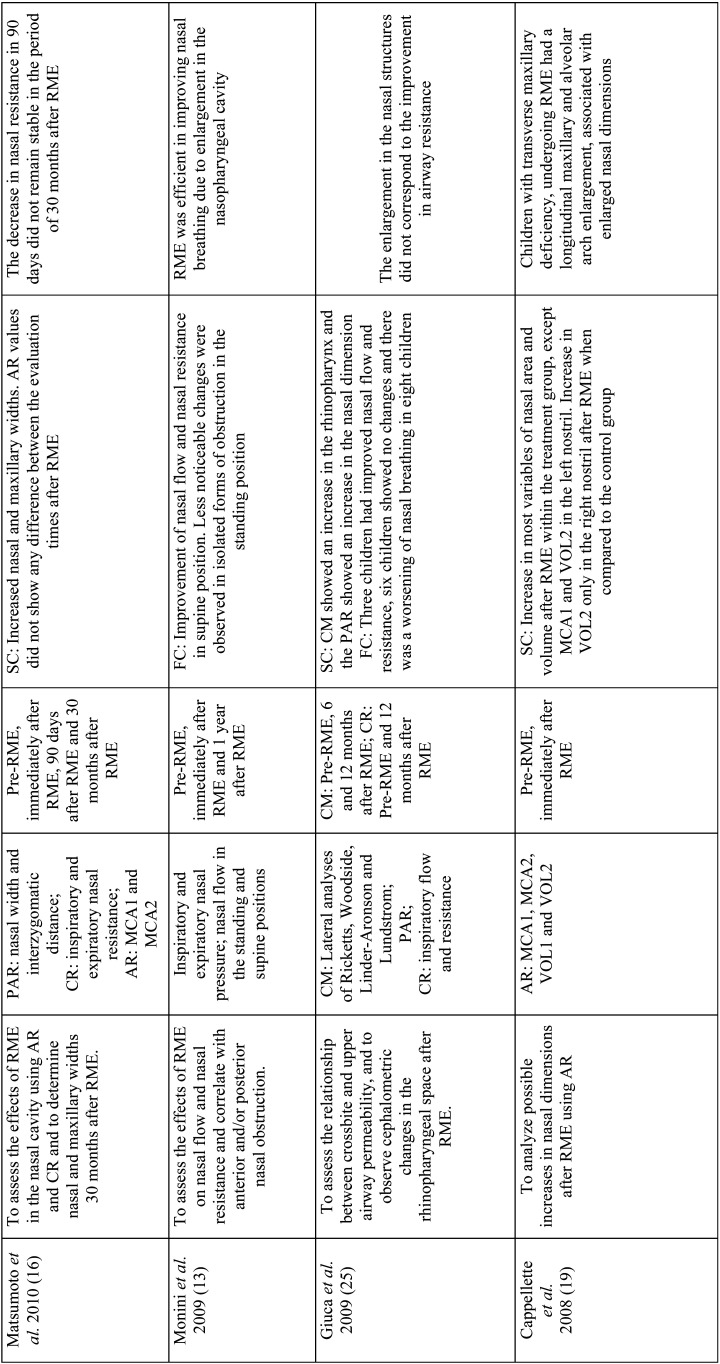
Analysis of objectives, variables, follow-up period, main results and conclusions.

**Table 2 cont.-2:**
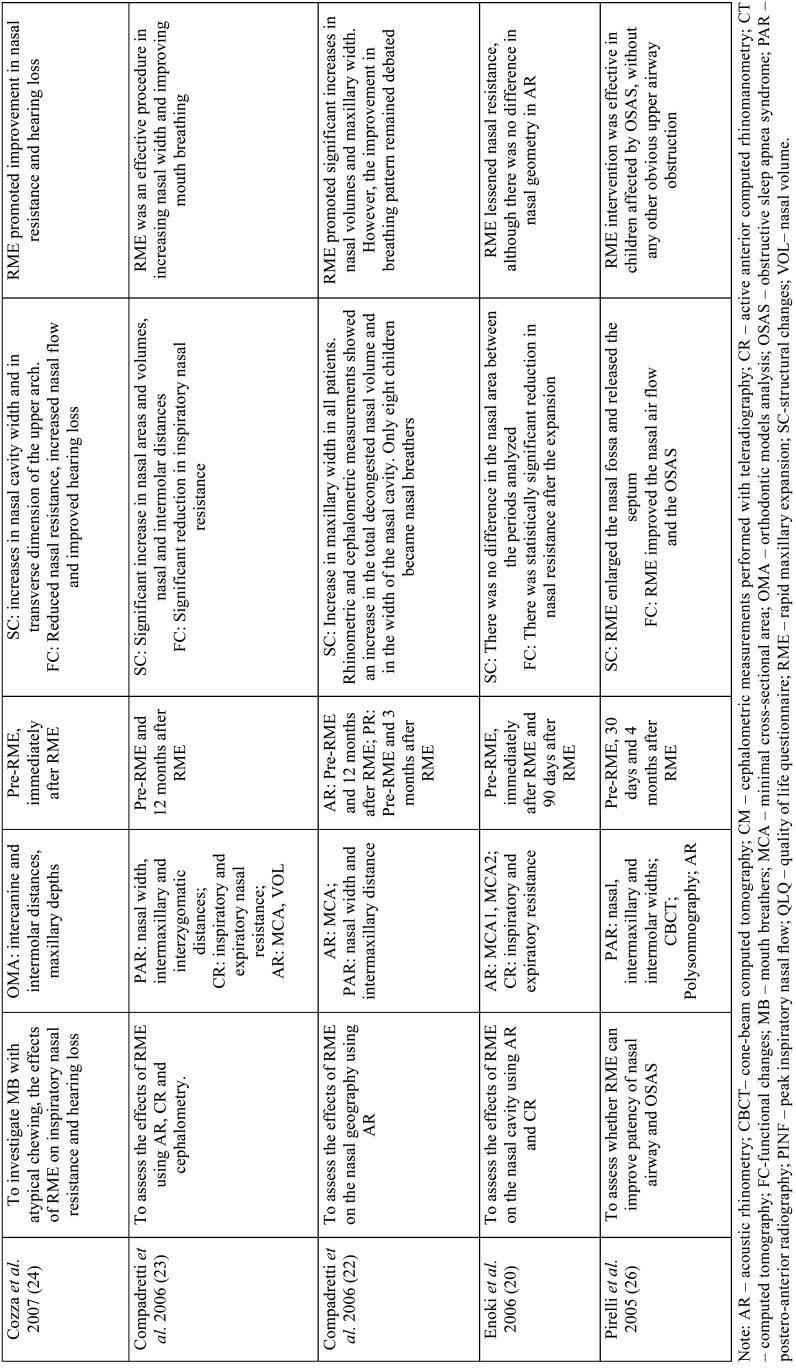
Analysis of objectives, variables, follow-up period, main results and conclusions.

-Evaluation of Methodological Quality of Studies

The quality of the studies was assessed by two evaluators, using the guidelines for appraisal of medical research proposed by Fowkes & Fulton (18). The questions associated with the evaluated parameters were scored as major (++), minor (+), absent (0) or not applicable (NA). Each study received an initial score of 46 points, with one parameter subtracted from each parameter (+) and two points from each parameter scored with (++). After this process, the evaluators proceeded to the final score, to classify the studies as: low quality (0–14), middle quality (15-30) and high quality (31-46) (Table 3).

**Table 3:**
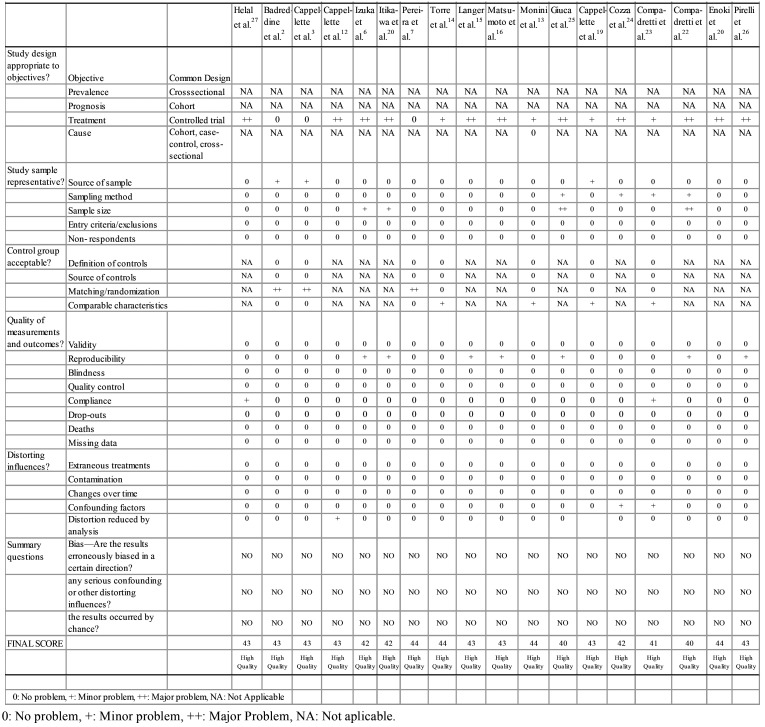
Analysis of the quality of the studies according to Fowkes and Fulton.

All studies analyzed in this systematic review were classified as controlled clinical trials, as they are experimental studies that used the RME orthodontic intervention ( Table 1). Fowkes and Fulton (16) proposes that in this type of study the results of the treatment group should be compared with another group with similar characteristics that have not received the intervention. Thus, the studies that did not include any comparison group received the score (++) in this first stage of assessment of methodological quality. Studies with a divergent comparison group of mouth breathers with TMD received a score (+).

Studies with a wide age range, which did not separately analyze groups of children with groups of adolescents, received a score (++) in the item source of sample.

Articles with less than 20 individuals and without a sample size calculation received a score (++) in the sample size item.

## Results

-Search and Selection

This systematic review identified 490 titles in the databases, making a total of 18 articles for this systematic review (Fig. 1).

The abstract screening process excluded articles that did not meet the pre-established criteria. For the full articles, one study was excluded as the language did not meet the eligibility criteria: the abstract was in English, but the full article was in Chinese. Other studies were also excluded due to inadequate methodology: retrospective studies, absence of diagnosis of mouth breathing, sample without history of respiratory diseases of the upper airways, nasal hypertrophy or respiratory allergies, absence of nasal respiratory function assessment after RME. Ultimately, one article was excluded because it included adults.

-Study characteristics

Most studies (10/18) were conducted in Brazil (2,3,6,12,15,19,20,21), six in Italy (13,22-26) , one in Spain (14) and one in the USA (27). The studies dated from 2005 to 2019 ( Table 1).

Only seven articles included a control group without the intervention of RME, but these varied according to the type of breathing (nasal or mouth) and as to the presence or absence of TMD ( Table 1). Four studies (2,3,7,19) included MB (three with TMD (2,3,7). Some studies (13,23,14) assessed nasal breathers (NB), one with TMD (23).

-Evaluated Instruments

The main instruments evaluated were: teleradiography (cephalometric measurements) (7,13,15,20,25), frontal postero-anterior radiography (nasal and maxillary widths) (3,16,21-computed tomography (three-dimensional airway measurements) (2,3), acoustic rhinometry (minimal cross-sectional area (MCA) and nasal volume (VOL)), computed rhinomanometry (respiratory resistance), analysis of orthodontic models (intercanine, intermolar and palate depth) and questionnaires on mouth breathing and quality of life (6). Only three studies evaluated using computed tomography scans (2,3,6) with only one using cone-beam computed tomography (6) (Table 2).

-Quality of Studies

The 18 selected studies were classified with high methodological quality ( Table 3) according to the appraisal guidelines proposed by Fowkes and Fulton (18).

## Discussion

In order to assess the effects of RME on naso-maxillary structures and nasal patency in MB as well as to verify the stability of these variables in the long term, the present review found that most studies (16/18) showed an increase in naso-maxillary structures (nasal cavity, oropharynx, nasopharynx, maxillary sinuses, maxillary width and dental arches) with RME (2,6,16,27). The effects on soft tissue structures of the nose were found on computed tomography scans three months after RME in the study by Badreddine *et al*. (2), for example.

Structural changes were detected by postero-anterior radiographic examinations, teleradiographs, conventional CT scans, cone-beam CT scans, dental arch models and acoustic rhinometry examinations (3,19,12,22,23).

In the study by Langer *et al*. (15), differences in nasopharyngeal space were found only after 30 months of RME, and could be explained by facial growth, and not because of the orthodontic procedure. In the study carried out by Enoki *et al*. (20), no statistically significant differences were observed in the measurements of the minimum cross-sectional area of the nasal valve and the inferior nasal concha with acoustic rhinometry, despite the improvement in nasal resistance with RME.

Functional changes were evaluated in eleven studies, through tests that provide objective data on nasal breathing such as active anterior computed rhinomanometry and peak inspiratory nasal flow, in addition to subjective tests. Among the studies analyzed, only Izuka *et al*. (6) and Helal *et al*. (27) used standardized questionnaires on respiratory patterns and symptoms, answered by the parents, showing improvement in the outcomes of the respiratory variables with RME.

Eight studies reported improvement in nasal respiratory function immediately after RME (6,13,14,21,22,24,26,). Monini *et al*. (13), in a study with 65 children submitted to RME compared to 50 children in the control group, found differences in nasal flow and resistance in the supine and orthostatic positions immediately and 12 months after RME. Likewise, Compadretti *et al*. (23) found a similar result in nasal resistance after the same follow-up period.

There was little evidence of absence of variation in respiratory flow after RME. In the study by Itikawa *et al*. (21), inspiratory nasal resistance returned to baseline values 90 days after RME. The same fact was observed by Matsumoto *et al*. (16), in the follow-up period of 30 months after RME, with the values of nasal resistance practically returned to baseline values. Such effects were related to the hypertrophied nasal mucosa, given that allergic rhinitis was the main cause of mouth breathing in these children.

Giuca *et al*. (25) reported a decrease in airway resistance in the active anterior rhinomanometry test in only three children, with six children showing no changes; airway resistance worsened in eight children during a follow-up period of 12 months after RME. In this study, no analysis was performed immediately after RME, showing an increase in structures. Therefore, no correlations were noted between nasal function and the RME procedure.

The enlargement of nasal structures with RME may cause an immediate improvement in breathing, but the persistence of the inflammatory process in the nasal mucosa is likely to favor the recurrence of hypertrophy of the nasal mucosa. When RME is indicated, it must be performed during the treatment or after treating the cause of the nasal obstruction (16).

For RME to be effective, it must be performed before the fusion of the medial palatal suture begins. Studies with cone-beam computed tomography found the beginning of the fusion of the median palatal suture in the palatal bone (a stage of sutural ossification called stage D (28)) in some female individuals at 11 years of age and male from 14 years of age. Therefore, in cases of RME, the structural bone age should be evaluated and not only the chronological age. Individuals who have advanced stages of sutural ossification may need complementary treatments such as RME, with the need for surgical intervention, either with micro-implant assisted rapid palatal expander (MARPE) or surgically assisted rapid maxillary expansion (SARPE) (28).

Imaging exams are important to assess the effects of RME on the medial palatine suture. CT scans allow better visualization of anatomical structures even in the presence of the palatal expander, minimizing the effects of radiographic artifacts (29).

In this review, the following orthodontic appliances were used: the Hyrax and modified Hyrax orthodontic appliances (14 studies) and the Hass and modified Hass appliances (4 studies). Also, the modified Biederman and Butterfly appliances were considered as modified Hyrax appliances.

The studies showed diverging results between the follow-up periods, namely, immediately after, one month, three, fourth, six, 12, 14 and 30 months after RME, showing temporal heterogeneity. Although methodologically adequate, they were not controlled and randomized.

Despite the adequate methodological quality, a great difference in the study methods used was observed. The present review showed that RME improves mouth breathing. On the other hand, there were no randomized controlled trials in MB with TMD, both in the short and long term, highlighting the need for further studies in this area.

## Conclusions

The use of ERM promotes the enlargement of the dental arches and of the nasal and maxillary structures, and improves mouth breathing in the short term. However, its long-term benefits have not been proved so far. More robust results of the effectiveness of RME with MB can be achieved in meta-analysis studies, with a consensual definition of the long-term follow-up period after RME.
